# 

**Published:** 1835

**Authors:** 


A WORD FROM THE PROPRIETORS.

The proprietors design publishing, at the close of the next number, the names of all those who have paid their subscriptions in advance. As the Journal has been irregular in its appearance in the hands of former proprietors, all payments made before the tenth of April shall be appended to the advance list. They are making arrangements for paper made expressly for the work, so that the entire volume will present a uniform appearance, both in letter and paper, as well as improving it in many other respects; consequently, the tenth volume will be attended with additional expense, and hence they hope their subscribers will pay in advance on that vol., at least. Three dollars is but a small sum to one man, while two thousand times that amount is of much importance to those who are daily paying large expenses. The editors and proprietors are determined that their work shall equal any other, both in matter and mechanical execution, in the Union. In future they expect to be responsible for the appearance of the work. Price 4 dollars at the close of the vol., 3 dollars and 50 cents at the end of three months, or 3 dollars in advance or within the first quarter.

JOHNSON & WOOD.

